# Target Tracking Using SePDAF under Ambiguous Angles for Distributed Array Radar

**DOI:** 10.3390/s16091456

**Published:** 2016-09-09

**Authors:** Teng Long, Honggang Zhang, Tao Zeng, Xinliang Chen, Quanhua Liu, Le Zheng

**Affiliations:** 1 Beijing Key Laboratory of Embedded Real-time Information Processing Technology, Radar Research Laboratory, School of Information and Electronics, Beijing Institute of Technology, Beijing 100081, China; longteng@bit.edu.cn (T.L.); zhanghg516@163.com (H.Z.); zengtao@bit.edu.cn (T.Z.); chenxinliang@bit.edu.cn (X.C.); 2 Electrical Engineering Department, Columbia University, New York, NY 10027, USA; lezheng8451@163.com

**Keywords:** distributed array radar, direction-of-arrival (DOA) estimation, ambiguous angles, tracking, probability data association filter (PDAF)

## Abstract

Distributed array radar can improve radar detection capability and measurement accuracy. However, it will suffer cyclic ambiguity in its angle estimates according to the spatial Nyquist sampling theorem since the large sparse array is undersampling. Consequently, the state estimation accuracy and track validity probability degrades when the ambiguous angles are directly used for target tracking. This paper proposes a second probability data association filter (SePDAF)-based tracking method for distributed array radar. Firstly, the target motion model and radar measurement model is built. Secondly, the fusion result of each radar’s estimation is employed to the extended Kalman filter (EKF) to finish the first filtering. Thirdly, taking this result as prior knowledge, and associating with the array-processed ambiguous angles, the SePDAF is applied to accomplish the second filtering, and then achieving a high accuracy and stable trajectory with relatively low computational complexity. Moreover, the azimuth filtering accuracy will be promoted dramatically and the position filtering accuracy will also improve. Finally, simulations illustrate the effectiveness of the proposed method.

## 1. Introduction

Distributed array radar has been widely concerned with its superiority in many aspects since it has been put forward [1]. For instance, owing to the waveform diversity and spatial diversity of the target’s radar cross-section (RCS), multi-input multi-output (MIMO) radar can obtain a diversity gain for target detection and for estimation of various parameters [2,3,4]. In addition, the distributed coherent aperture radar has been proposed in order to obtain the *N*^3^ times signal-to-noise ratio (SNR) promotion, where *N* is the number of sub-radars and, meanwhile, avoid the difficult transportability, high cost, and other drawbacks of large aperture radar [5,6].

For the traditional radar, target tracking is employed after achieving the target’s direction-of-arrival (DOA) estimation, which has been widely studied [7,8,9]. The Kalman filter (KF), extended Kalman filter (EKF), and other more complicated filters, can be utilized for tracking [10]. However, the distributed array radar will suffer cyclic ambiguity in its angle estimates according to the spatial Nyquist sampling theorem since the large sparse array is undersampling. In results, the state estimation accuracy and track validity probability will be very poor when the ambiguous angles are directly used for target tracking.

In order to resolve this problem, distributed array radar currently applies the traditional radar’s tracking mode which, firstly, estimates the high accuracy angle without ambiguity, then tracks the target. There are three kinds of methods to achieve the high accuracy DOA estimation for a distributed array. The first kind is designing a low sidelobe sparse array, thus avoiding the ambiguous angles coming from the grating lobes. For instance, the array interelement spacing is optimized to achieve an objective function with a narrow peak around the true target parameters and the lowest possible sidelobes at all other parameter combinations, thus achieving the combined range and angle estimation for frequency modulated continuous wave (FMCW) radar sensors [11]. A new method of estimating the DOA for multiple signals using minimum redundancy linear sparse subarrays (MRLSS) is proposed [12,13].

The second kind uses two different arrays to obtain the unambiguous, but high-variance, direction estimate and low-variance, but cyclically ambiguous, estimates, respectively, then the unambiguous estimate serves as a coarse reference to disambiguate the set of low variance ambiguous estimates. The dual-size spatial invariance sparse array concept for estimation of signal parameters via a rotational invariance techniques (ESPRIT)-based algorithm, which is capable of extending the array aperture without any cyclic ambiguity in the final DOA estimates is introduced [14]. A multiple signal classification (MUSIC) or method of direction estimation (MODE)-based algorithm which improves and generalizes disambiguation schemes that populate the thin array grid with identical subarrays is proposed [15]. A new total least squares ESPRIT (TLS-ESPRIT)-based DOA estimator is obtained for the sparse sensor arrays with multiple identical subarrays [16]. A resultant algorithm which is the combination of the ESPRIT algorithm with MUSIC (or conventional beamformer) and permits the identification of the true direction cosine estimates from a set of ambiguous candidate estimates is presented [17]. The coarse estimates are used to disambiguate the fine, but ambiguous, estimates of direction cosines which are achieved by utilizing the inter-sensor spacing phase-factors in the sparse array [18]. A method which divided the linear sparse arrays into two overlapping subarrays, then exploited the ESPRIT-like algorithm to get DOA estimates is contrived [19].

The third kind firstly uses compressed sensing to recover the filled array data from thin array data, then applies the angle estimation method to achieve the unambiguous estimate. Two sparse recovery methods based on different optimization problems are proposed to solve the DOA estimation problem in the sparse array [20]. The problem of joint DOA estimation with distributed sparse linear arrays is presented and an off-grid synchronous approach based on distributed compressed sensing is proposed [21]. A two-dimensional (2D) DOA estimation algorithm is proposed with the co-prime array based on the sparse representation framework [22]. An off-grid DOA estimation method using sparse Bayesian learning (SBL) based on an array covariance matrix is presented [23]. Without the knowledge of the number of sources, these methods yield superior performances.

However, although the grating lobes can be suppressed effectively, the mainlobe will widen and the sidelobes will rise inevitably for the first method. Consequently, the angle estimation accuracy will decrease. The second method will produce a failure result if the unambiguous angle fails to disambiguate the ambiguous angles, leading to a bias from the true angle. The third method may become invalid when the inter-arrays spacing is large, and the computational burden is expensive due to the compressed sensing. Therefore, using these results directly for the tracking filter may result in a reduction of the state estimation accuracy, or even stable tracking.

Inspired by the DOA tracking method [24] and the multiple sensors tracking method [25,26,27,28,29,30], this paper proposes a second probability data association filter (SePDAF)-based tracking method. It uses the unambiguous angle and ambiguous angles as measurements, then twice applying filtering, i.e., EKF and SePDAF, to achieve the high accuracy unambiguous filtering estimate and stable trajectory simultaneously. This method produces a novel tracking mode with relatively low computational complexity for distributed array radar in order to replace the traditional one. This paper is organized as follows: Section 1 introduces the distributed array radar and its tracking under ambiguous angles; Section 2 is the signal model, and it builds the target motion model and distributed array radar measurement model; Section 3 firstly analyzes the probability model, then proposes the SePDAF method to achieve the high accuracy trajectory, after that investigates the computational complexity; Section 4 carries out the simulations to validate the effectiveness of the proposed method; and, finally, Section 5 draws the conclusion.

## 2. Signal Model

In radar tracking, the measurements of a target’s position are generally expressed in three-dimensional (3D) polar coordinates, while the target motion is usually precisely modeled in Cartesian coordinates.

### 2.1. Target Motion Model

According to the target motion, different kinds of motion models can be utilized. For instance, a constant velocity motion model can be employed when the target velocity is constant [10,26]; constant acceleration motion model can be used when the target has a uniformly variable velocity [10]; ellipsoidal Earth model is generally utilized to precisely describe the gravity of the Earth for space target tracking [31], etc.

For simplicity, the constant velocity target motion model is assumed in this paper. The proposed algorithm here is suitable for other target motion models. The state equation of constant velocity motion is:

(1) x(k+1)=F(k)x(k)+v(k)

where, *k* is sampling time, and it is also called slow time, the state vector at time *k* is $x(k)={\left[\begin{array}{cccccc} x(k) & \dot{x}(k) & y(k) & \dot{y}(k) & z(k) & \dot{z}(k) \end{array}\right]}^{T}$, x(k), y(k), z(k) denotes the target position, $\dot{x}(k),\;\dot{y}(k),\;\dot{z}(k)$ denotes the target velocity, and the superscript *^T^* means transpose. F(k) is the state transition matrix, v(k) is zero-mean white Gaussian process noise vector with non-singular covariance matrix Q(k). If $\Gamma (k)\tilde{v}(k)$ is used to replace v(k), then Q(k) becomes $\Gamma (k)q\Gamma {\left(k\right)}^{T}$, where *q* means the variance of process noise $\sigma_{v}^{2}$, and Γ(k) is the process noise distribution matrix. The state transition matrix and process noise distribution matrix are:

(2) $$F\left(k\right)=\left[\begin{array}{cccccc} 1 & T & 0 & 0 & 0 & 0\\ 0 & 1 & 0 & 0 & 0 & 0\\ 0 & 0 & 1 & T & 0 & 0\\ 0 & 0 & 0 & 1 & 0 & 0\\ 0 & 0 & 0 & 0 & 1 & T\\ 0 & 0 & 0 & 0 & 0 & 1 \end{array}\right]\;\;\;\;\Gamma \left(k\right)=\left[\begin{array}{ccc} 0.5T^{2} & 0 & 0\\ T & 0 & 0\\ 0 & 0.5T^{2} & 0\\ 0 & T & 0\\ 0 & 0 & 0.5T^{2}\\ 0 & 0 & T \end{array}\right]$$

where *T* is the sampling time interval.

### 2.2. Radar Measurement Model

#### 2.2.1. Radar Receiving Signal

Assume the distributed array radar consists of *N* sub-radars which are arranged in a uniform linear array (ULA), the sub-radar spacing is *d*, thus the baseline of the distributed array is (*N*−1)*d*. Each sub-radar is also a ULA which consists of *M* antenna elements. Suppose the complex envelope of the signal reflected from the target is *p*(*t*), where *t* is fast time, then the complex envelope of the signal arriving at the *n*th radar becomes $p[t-\tau_{n}(k)]$, where $\tau_{n}(k)$ is the time delay between the target and the *n*th radar at time *k*, and *n* = 1,2,…,*N*. Therefore, the signal can be written as a vector:

(3) $$s\left(k\right)={\left[\begin{array}{cccc} s_{1}\left(k\right) & s_{2}\left(k\right) & \cdots & s_{N}\left(k\right) \end{array}\right]}^{T}$$

where, $s_{n}(k)$ is the baseband signal after digital down converter of the *n*th radar, which is:

(4) $$\begin{array}{ll} s_{n}\left(k\right) & =\exp\left[-j2\pi f_{c}\left(k\right)\tau_{n}\left(k\right)\right]p\left[t-\tau_{n}\left(k\right)\right]\\ & \approx \exp\left[-j2\pi f_{c}\left(k\right)\frac{r_{1}\left(k\right)+\left(n-1\right)d\sin\theta \left(k\right)}{c}\right]p\left[t-\frac{r_{1}\left(k\right)}{c}\right] \end{array}$$

where, $j=\sqrt{-1}$ and $f_{c}(k)$ is the carrier frequency at time *k*. It means that the carrier frequency can be staggered at a different time. $r_{1}(k)$ is the distance between the target and the first radar at time *k*, θ(k) is the target direction at time *k*, *c* is the speed of light. Considering the receiver noise, we have the receiving signal vector:

(5) y(k)=s(k)+n(k)

where, n(k) is the noise vector at time *k*, and each sub-radars’ noise are all zero-mean white Gaussian noise.

#### 2.2.2. Radar Measurement Accuracy

For distributed array radar, the range, azimuth, and elevation angle measurements of each radar can be fused to achieve higher accuracy estimates. Moreover, the high accuracy, but cyclically ambiguous, azimuth estimates can be obtained by using the large baseline sparse array. The accuracies of these radar measurements are discussed here.

Firstly, the range estimation accuracy is analyzed. Applying the traditional range estimation method to each sub-radar, we can get the range estimate ${\hat{r}}_{n}(k)$ of the *n*th radar at time *k*, and its variance, i.e., accuracy, is:

(6) $$\sigma_{r_{n}}^{2}\left(k\right)={\left[\frac{c}{4B{\left(L{\left(S/N\right)}_{n}\right)}^{1/2}}\right]}^{2}$$

where, *B* is the signal bandwidth, *L* is the snapshot number, (*S*/*N*)*_n_* is the SNR of the *n*th radar. Then the range estimates can be averaged by variable weight, the range fusion result and its corresponding accuracy are:

(7) $$\hat{r}\left(k\right)={\left[\sum_{n=1}^{N}\frac{1}{\sigma_{r_{n}}^{2}\left(k\right)}\right]}^{-1}\left[\sum_{n=1}^{N}\frac{1}{\sigma_{r_{n}}^{2}\left(k\right)}{\hat{r}}_{n}\left(k\right)\right]$$

(8) $$\sigma_{r}^{2}\left(k\right)={\left[\sum_{n=1}^{N}\frac{1}{\sigma_{r_{n}}^{2}\left(k\right)}\right]}^{-1}$$

Secondly, the azimuth and elevation angle estimation fusion accuracies are analyzed. Since their analysis processes are the same, we take the azimuth as an example. Applying the traditional angle estimation method to each sub-radar, we can get the azimuth estimate ${\hat{\theta }}_{n}^{s}(k)$ of the *n*th radar at time *k*, and its variance is:

(9) $$\sigma_{\theta_{n}^{s}}^{2}\left(k\right)={\left[\frac{c}{K_{m}f_{c}\left(k\right)D{\left(L{\left(S/N\right)}_{n}\right)}^{1/2}}\right]}^{2}$$

where, *K_m_* is the slope of the angle error, *D* is the aperture of sub-radar. Averaging these estimates by different weight, the azimuth fusion result and its corresponding accuracy can be obtained, which can be expressed as:

(10) $${\hat{\theta }}^{s}\left(k\right)={\left[\sum_{n=1}^{N}\frac{1}{\sigma_{\theta_{n}^{s}}^{2}\left(k\right)}\right]}^{-1}\left[\sum_{n=1}^{N}\frac{1}{\sigma_{\theta_{n}^{s}}^{2}\left(k\right)}{\hat{\theta }}_{n}^{s}\left(k\right)\right]$$

(11) $$\sigma_{\theta^{s}}^{2}\left(k\right)={\left[\sum_{n=1}^{N}\frac{1}{\sigma_{\theta_{n}^{s}}^{2}\left(k\right)}\right]}^{-1}$$

Thirdly, taking advantage of the large baseline of distributed array radar, the high accuracy azimuth estimation can be achieved. Suppose there is no ambiguity in the estimation, thus the azimuth estimation can be written as:

(12) $${\hat{\theta }}^{u}\left(k\right)=\theta \left(k\right)+w_{\theta }\left(k\right)$$

where, $w_{\theta }(k)$ is the zero-mean white Gaussian noise at time *k*, i.e., $w_{\theta^{u}}(k)\sim N(0,\sigma_{\theta^{u}}^{2}(k))$. The accuracy of the array processed azimuth estimation is approximate to:

(13) $$\sigma_{\theta^{u}}^{2}\left(k\right)={\left[\frac{c}{K_{m}f_{c}\left(k\right)\left(N-1\right)d{\left(LN{\left(S/N\right)}_{n}\right)}^{1/2}}\right]}^{2}$$

It can be seen that this accuracy is very high since the distributed array has a large baseline (*N*−1)*d*. Unfortunately, the array processed azimuth estimation has numerous ambiguous estimates due to the thin array. Assume there are *m_k_* ambiguous azimuth angles, and these ambiguous ones are symmetrical about the true estimate. Then the array processed azimuth estimation can be modified as:

(14) $${\hat{\theta }}_{i}^{u}\left(k\right)=\{\begin{array}{cc} \arcsin\left[\sin{\hat{\theta }}^{u}\left(k\right)+\frac{c}{f_{c}\left(k\right)d}\cdot \left(i-\frac{m_{k}}{2}\right)\right], & m_{k}\;\mathrm{is}\;\mathrm{even}\\ \arcsin\left[\sin{\hat{\theta }}^{u}\left(k\right)+\frac{c}{f_{c}\left(k\right)d}\cdot \left(i-\frac{m_{k}+1}{2}\right)\right], & m_{k}\;\mathrm{is}\;\mathrm{odd} \end{array}$$

where *i* = 1,2,…,*m_k_*.

#### 2.2.3. Radar Measurement Model

The measurement equation can then be expressed as:

(15) z(k)=h[x(k)]+w(k)

where, the measurement vector is $z(k)={\left[\begin{array}{ccc} \hat{r}(k) & \hat{\theta }(k) & \hat{\varphi }(k) \end{array}\right]}^{T}$, and $\hat{r}(k),\;\hat{\theta }(k),\;\hat{\varphi }(k)$ are the measurements of the range, azimuth, and elevation angle. w(k) is a zero-mean white Gaussian measurement noise vector which is independent with the process noise v(k). Its covariance matrix is:

(16) $$R\left(k\right)=\mathrm{diag}\left(\sigma_{r}^{2},\sigma_{\theta }^{2},\sigma_{\varphi }^{2}\right)$$

where diag(⋅) stands for a diagnose matrix, $\sigma_{r}^{2},\;\sigma_{\theta }^{2},\;\sigma_{\varphi }^{2}$ are the variances of the range, azimuth, and elevation angle measurements. In addition, the measurement function is given as:

(17) $$h\left[x\left(k\right)\right]=\left[\begin{array}{c} \sqrt{x^{2}\left(k\right)+y^{2}\left(k\right)+z^{2}\left(k\right)}\\ \tan^{-1}\left[y\left(k\right)/x\left(k\right)\right]\\ \tan^{-1}\left[z\left(k\right)/\sqrt{x^{2}\left(k\right)+y^{2}\left(k\right)}\right] \end{array}\right]$$

where the Jacobian matrix can be written as:

(18) $$H_{x}\left(k\right)=\left[\begin{array}{cccccc} \frac{x\left(k\right)}{\rho \left(k\right)} & 0 & \frac{y\left(k\right)}{\rho \left(k\right)} & 0 & \frac{z\left(k\right)}{\rho \left(k\right)} & 0\\ \frac{-y\left(k\right)}{{\rho_{xy}}^{2}\left(k\right)} & 0 & \frac{x\left(k\right)}{\rho_{xy}^{2}\left(k\right)} & 0 & 0 & 0\\ \frac{-x\left(k\right)z\left(k\right)}{\rho_{xy}\left(k\right)\cdot \rho^{2}\left(k\right)} & 0 & \frac{-y\left(k\right)z\left(k\right)}{\rho_{xy}\left(k\right)\cdot \rho^{2}\left(k\right)} & 0 & \frac{\rho_{xy}\left(k\right)}{\rho^{2}\left(k\right)} & 0 \end{array}\right]$$

and:

(19) $$\rho \left(k\right)=\sqrt{x^{2}\left(k\right)+y^{2}\left(k\right)+z^{2}\left(k\right)}$$

(20) $$\rho_{xy}\left(k\right)=\sqrt{x^{2}\left(k\right)+y^{2}\left(k\right)}$$

When the azimuth fusion result ${\hat{\theta }}^{s}(k)$ is used as the azimuth measurement, the measurement vector becomes $z^{s}(k)={\left[\begin{array}{ccc} \hat{r}(k) & {\hat{\theta }}^{s}(k) & \hat{\varphi }(k) \end{array}\right]}^{T}$, and the covariance matrix of the measurement noise becomes $R^{s}(k)=\mathrm{diag}(\sigma_{r}^{2},\sigma_{\theta^{s}}^{2},\sigma_{\varphi }^{2})$, where $\sigma_{\theta^{s}}^{2}$ denotes the azimuth fusion accuracy.

On the other hand, when the array processed azimuth estimation ${\hat{\theta }}_{i}^{u}(k)$ is used, the measurement vector changes to $z_{i}^{u}(k)={\left[\begin{array}{ccc} \hat{r}(k) & {\hat{\theta }}_{i}^{u}(k) & \hat{\varphi }(k) \end{array}\right]}^{T}$, where *i* denotes the *i*th measurement vector out of *m_k_*. The corresponding measurement noise covariance matrix changes to $R^{u}(k)=\mathrm{diag}(\sigma_{r}^{2},\sigma_{\theta^{u}}^{2},\sigma_{\varphi }^{2})$, where $\sigma_{\theta^{u}}^{2}$ means the array processed azimuth accuracy.

## 3. Second Probability Data Association Filter-Based Tracking

### 3.1. Probability Model

Assume the state vector estimation at time k−1 is $\hat{x}(k-1|k-1)$, the corresponding covariance matrix is P(k−1|k−1). Suppose the real state vector is x(k−1|k−1), then the probability density function is:

(21) $$p\left(\hat{x}\left(k-1|k-1\right)|\Theta^{k-1}\right)=\frac{1}{\sqrt{\left|2\pi P\left(k-1|k-1\right)\right|}}\times \exp\left\{\begin{array}{l} -\frac{1}{2}{\left[\hat{x}\left(k-1|k-1\right)-x\left(k-1|k-1\right)\right]}^{T}\\ \times P^{-1}\left(k-1|k-1\right)\left[\hat{x}\left(k-1|k-1\right)-x\left(k-1|k-1\right)\right] \end{array}\right\}$$

where, $\Theta^{k-1}$ denotes the observation set from the beginning to time k−1. The observation set at time *k* can be written as $\Theta (k)=\{\Theta^{u}(k),\Theta^{s}(k)\}$, where $\Theta^{u}(k)$ represent the array processed measurement set and $\Theta^{s}(k)$ represents the fusion measurement set.

As for the observation set at time *k*, suppose $z^{u}(k)$ is the true estimate of target from the ambiguous measurements $z_{i}^{u}(k)$, x(k) is the true state vector. Then the probability density function can be expressed as:

(22) $$p\left(z^{u}\left(k\right)|h\left[x\left(k\right)\right]\right)=\frac{1}{\sqrt{\left|2\pi R^{u}\left(k\right)\right|}}\exp\left\{\begin{array}{l} -\frac{1}{2}{\left[z^{u}\left(k\right)-h\left[x\left(k\right)\right]\right]}^{T}\\ \times R^{u}{\left(k\right)}^{-1}\left[z^{u}\left(k\right)-h\left[x\left(k\right)\right]\right] \end{array}\right\}$$

As is known, there are *m_k_* cyclic ambiguous azimuths in the array processed measurement, then let the *i*th measurement producing from the true target be the event A(k)=i. Therefore, the probability density function becomes:

(23) $$p\left(A\left(k\right)=i|h\left[x\left(k\right)\right]\right)=\frac{1}{m_{k}}$$

(24) $$p\left(z_{i}^{u}\left(k\right)|A\left(k\right)=i,h\left[x\left(k\right)\right]\right)=\frac{1}{\sqrt{\left|2\pi R^{u}\left(k\right)\right|}}\exp\left\{\begin{array}{l} -\frac{1}{2}{\left[z_{i}^{u}\left(k\right)-h\left[x\left(k\right)\right]\right]}^{T}\\ \times R^{u}{\left(k\right)}^{-1}\left[z_{i}^{u}\left(k\right)-h\left[x\left(k\right)\right]\right] \end{array}\right\}$$

However, the target true state usually cannot be obtained in practice. We can only focus on the conditional probability based on predicted values. Thus, the probability density function can be expressed as:

(25) $$p\left(A\left(k\right)=i|\Theta^{k-1}\right)=\frac{1}{m_{k}}$$

(26) $$\begin{array}{l} p\left(\Theta^{u}\left(k\right)|A\left(k\right)=i,\Theta^{k-1}\right)=p\left(z_{i}^{u}\left(k\right)|A\left(k\right)=i,\Theta^{k-1}\right)\\ \;\;\;\;\;\;\;=\frac{1}{\sqrt{\left|2\pi S^{u}\left(k\right)\right|}}\exp\left\{\begin{array}{l} -\frac{1}{2}{\left[z_{i}^{u}\left(k\right)-h\left[{\hat{x}}^{u}\left(k|k-1\right)\right]\right]}^{T}\\ \times S^{u}{\left(k\right)}^{-1}\left[z_{i}^{u}\left(k\right)-h\left[{\hat{x}}^{u}\left(k|k-1\right)\right]\right] \end{array}\right\} \end{array}$$

where, ${\hat{x}}^{u}(k|k-1)$ is the predicted state vector and $S^{u}(k)$ is the covariance matrix of innovation.

### 3.2. Second Probability Data Association Filter

In order to achieve target tracking under ambiguous angles for distributed array radar, the second probability data association filter-based tracking method is proposed. The fusion measurement is firstly used to accomplish the first filtering, then taking this result as prior knowledge, the array processed ambiguous measurement is utilized to complete the second filtering. The recursive processes from time k−1 to *k* are derived as follows:

#### 3.2.1. First Filtering

Assume that the state vector estimate and covariance matrix at time k−1 are ${\hat{x}}^{u}(k-1|k-1)$ and $P^{u}(k-1|k-1)$, respectively. With the EKF method, the state vector and the covariance matrix are predicted as:

(27) $${\hat{x}}^{u}\left(k|k-1\right)=F\left(k-1\right){\hat{x}}^{u}\left(k-1|k-1\right)$$

(28) $$P^{u}\left(k|k-1\right)=F\left(k-1\right)P^{u}\left(k-1|k-1\right)F^{T}\left(k-1\right)+Q\left(k\right)$$

The measurement vector prediction and its corresponding covariance matrix, and the gain matrix of EKF are given as:

(29) $${\hat{z}}^{u}\left(k|k-1\right)=h\left[{\hat{x}}^{u}\left(k|k-1\right)\right]$$

(30) $$S^{s}\left(k\right)=H_{x}\left[k,{\hat{x}}^{u}\left(k|k-1\right)\right]P^{u}\left(k|k-1\right){H_{x}}^{T}\left[k,{\hat{x}}^{u}\left(k|k-1\right)\right]+R^{s}\left(k\right)$$

(31) $$W^{s}\left(k\right)=P^{u}\left(k|k-1\right){H_{x}}^{T}\left[k,{\hat{x}}^{u}\left(k|k-1\right)\right]{\left[S^{s}\left(k\right)\right]}^{-1}$$

The updated state vector estimate and the corresponding covariance matrix of the first filtering are then calculated using the fusion measurement $z^{s}(k)$ as:

(32) $${\hat{x}}^{s}\left(k|k\right)={\hat{x}}^{u}\left(k|k-1\right)+W^{s}\left(k\right)\left[z^{s}\left(k\right)-{\hat{z}}^{u}\left(k|k-1\right)\right]$$

(33) $$P^{s}\left(k|k\right)=P^{u}\left(k|k-1\right)-W^{s}\left(k\right)S^{s}\left(k\right){\left[W^{s}\left(k\right)\right]}^{T}$$

The probability density function of the first filtering state estimate is:

(34) $$p\left({\hat{x}}^{s}\left(k|k\right)|\Theta^{s}(k),\Theta^{k-1}\right)=\frac{1}{\sqrt{\left|2\pi P^{s}\left(k|k\right)\right|}}\exp\left\{\begin{array}{l} -\frac{1}{2}{\left[{\hat{x}}^{s}\left(k|k\right)-x\left(k|k\right)\right]}^{T}\\ \times P^{s}{\left(k|k\right)}^{-1}\left[{\hat{x}}^{s}\left(k|k\right)-x\left(k|k\right)\right] \end{array}\right\}$$

where x(k|k) is the target true state vector at time *k*. After the above process, the first filtering is accomplished.

#### 3.2.2. Second Filtering

The above state estimate and the corresponding covariance matrix are taken as prior knowledge. The array processed measurement is then utilized to complete the second filtering. The predicted measurement vector, innovation covariance matrix, and gain matrix after the first filtering are calculated as:

(35) $${\hat{z}}^{s}\left(k|k\right)=h\left[{\hat{x}}^{s}\left(k|k\right)\right]$$

(36) $$S^{u}\left(k\right)=H_{x}\left[k,{\hat{x}}^{s}\left(k|k\right)\right]P^{s}\left(k|k\right){H_{x}}^{T}\left[k,{\hat{x}}^{s}\left(k|k\right)\right]+R^{u}\left(k\right)$$

(37) $$W^{u}\left(k\right)=P^{s}\left(k|k\right){H_{x}}^{T}\left[k,{\hat{x}}^{s}\left(k|k\right)\right]{\left[S^{u}\left(k\right)\right]}^{-1}$$

The association probability of each ambiguous estimate is calculated. Let the *i*th measurement $z_{i}^{u}(k)$ producing from the true target be the event *A*(*k*) = *i*, then the conditional probability of this event is:

(38) $$\begin{array}{ll} \beta_{i}\left(k\right) & =\mathrm{Pr}\left\{A\left(k\right)=i|\Theta^{k}\right\}=\mathrm{Pr}\left\{A\left(k\right)=i|\Theta^{u}\left(k\right),\Theta^{s}\left(k\right),\Theta^{k-1}\right\}\\ & =\frac{p\left(A\left(k\right)=i,\Theta^{u}\left(k\right)|\Theta^{s}\left(k\right),\Theta^{k-1}\right)}{p\left(\Theta^{u}\left(k\right)|\Theta^{s}\left(k\right),\Theta^{k-1}\right)}\\ & =\frac{p\left(A\left(k\right)=i|\Theta^{s}\left(k\right),\Theta^{k-1}\right)p\left(\Theta^{u}\left(k\right)|A\left(k\right)=i,\Theta^{s}\left(k\right),\Theta^{k-1}\right)}{\sum_{j=1}^{m_{k}}\left[p\left(A\left(k\right)=j|\Theta^{s}\left(k\right),\Theta^{k-1}\right)p\left(\Theta^{u}\left(k\right)|A\left(k\right)=j,\Theta^{s}\left(k\right),\Theta^{k-1}\right)\right]} \end{array}$$

where:

(39) $$p\left(A\left(k\right)=i|\Theta^{s}\left(k\right),\Theta^{k-1}\right)=p\left(A\left(k\right)=i|\Theta^{k-1}\right)=\frac{1}{m_{k}}$$

(40) $$\begin{array}{ll} p(\Theta^{u}\left(k\right)|A\left(k\right) & =i,\Theta^{s}\left(k\right),\Theta^{k-1})=p\left(z_{i}^{u}\left(k\right)|A\left(k\right)=i,\Theta^{s}\left(k\right),\Theta^{k-1}\right)\\ & =\frac{1}{\sqrt{\left|2\pi S^{u}\left(k\right)\right|}}\exp\left\{\begin{array}{l} -\frac{1}{2}{\left[z_{i}^{u}\left(k\right)-{\hat{z}}^{s}\left(k|k\right)\right]}^{T}\\ \times S^{u}{\left(k\right)}^{-1}\left[z_{i}^{u}\left(k\right)-{\hat{z}}^{s}\left(k|k\right)\right] \end{array}\right\} \end{array}$$

The updated state vector estimate and the corresponding covariance matrix of the second filtering can be acquired as:

(41) $$\begin{array}{ll} {\hat{x}}^{u}\left(k|k\right) & ={\hat{x}}^{s}\left(k|k\right)+W^{u}\left(k\right)\sum_{i=1}^{m_{k}}\beta_{i}\left(k\right)v_{i}^{u}\left(k\right)\\ & ={\hat{x}}^{s}\left(k|k\right)+W^{u}\left(k\right)v^{u}\left(k\right) \end{array}$$

(42) $$\begin{array}{ll} P^{u}\left(k|k\right)= & P^{s}\left(k|k\right)-W^{u}\left(k\right)S^{u}\left(k\right){\left[W^{u}\left(k\right)\right]}^{T}\\ & +W^{u}\left(k\right)\left\{\sum_{i=1}^{m_{k}}\beta_{i}\left(k\right)v_{i}^{u}\left(k\right){\left[v_{i}^{u}\left(k\right)\right]}^{T}-v^{u}\left(k\right){\left[v^{u}\left(k\right)\right]}^{T}\right\}{\left[W^{u}\left(k\right)\right]}^{T} \end{array}$$

where the innovation vector can be computed as:

(43) $$v_{i}^{u}\left(k\right)=z_{i}^{u}\left(k\right)-{\hat{z}}^{s}\left(k|k\right),i=1,2,...,m_{k}$$

The stable target tracking under ambiguous azimuth angles for distributed array radar can be successfully completed after employing the aforementioned steps. As a result, the azimuth filtering accuracy will be promoted significantly and the position filtering accuracy will also improve.

### 3.3. Computational Complexity

In this section, the computational complexity of the proposed method is investigated and compared with the EKF method. Since these algorithms can be decomposed into basic mathematical operations whose computational complexities are well known, such as matrix multiplication, matrix inversion, etc., the computational complexity analysis based on these operations can be shown in Table 1.

The first column lists the mathematical operations, here A and B are matrices or vectors whose size are denoted by the subscript, *N* is the order of state vector, α is a constant value, $\beta_{n}$ and $\gamma_{n}$ are two numbers where *n* refers to the number of digits. In our paper, the constant velocity motion model is used, thus *N* = 6. If the constant acceleration motion model is used, then *N* = 9. It can be seen that *N* is a small value. The size of measurement vector is three, which is less than *N*; for simplicity, we treat it as *N* in order to reduce the number of operations. The second column lists the computational complexities of these operations. Then each operation has been counted for the two algorithms, i.e., EKF and SePDAF, and their numbers are listed in the third and fourth columns. The fifth column is the counting number of SePDAF divided by the counting number of EKF under different complexity operations.

It can be found that compared with EKF, SePDAF almost has the same number of $O(N^{3})$ operations whose complexity is the highest; while for the other lower complexity operations, SePDAF have no more than 3*m_k_* times the counting number over EKF. Since *m_k_* is a finite number and it can be sharply reduced by introducing the fusion estimation as prior knowledge, the computational complexity of the lower complexity operations can also be greatly decreased. To sum up, since $O(N^{3})$ operations have the highest computational complexity, we can conclude that our proposed SePDAF will almost have the same computation time compared with EKF. Furthermore, the proposed two step filtering will double the computation time. Fortunately, this is an acceptable cost in reality in order to achieve higher accuracy azimuth estimation. Therefore, we can conclude that our proposed method has relatively low computational complexity.

## 4. Simulations

**Figure 1:**
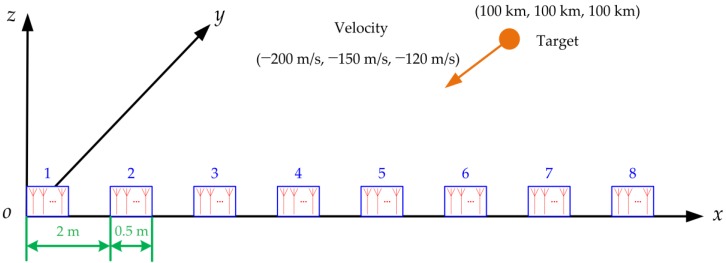
Diagram of the simulation scenario.

The diagram of the simulation scenario is shown in Figure 1 and the simulation parameters are shown in Table 2. The distributed array radar works in the C-band, it consists of eight identical sub-radars and it arranges in ULA whose sub-radar spacing is 2 m, so the baseline is 14 m. The sub-radar is also ULA with 20 antenna elements, and its antenna aperture is 0.5 m. The reference radar is positioned at the origin of the Cartesian coordinate system, the initial target position in 3D Cartesian coordinate is (100 km, 100 km, 100 km), the target constant velocity in 3D Cartesian coordinate is (−200 m/s, −150 m/s, −120 m/s), where each part of the 3D Cartesian coordinate denotes the projection in the *x*-axis, *y*-axis, and *z*-axis. The carrier frequency of the transmitting signal is 6 GHz, the bandwidth is 10 MHz, and the snapshot number is 10. The variance of the process noise is 1. Assuming the SNR is 20 dB, the fusion measurement noise and array processed measurement noise are $R^{s}(k)=\mathrm{diag}[{\left(0.0838m\right)}^{2},{\left(0.0408^{{}^{\circ}}\right)}^{2},{\left(0.0408^{{}^{\circ}}\right)}^{2}]$ and $R^{u}(k)=\mathrm{diag}[{\left(0.0838m\right)}^{2},{\left(0.0013^{{}^{\circ}}\right)}^{2},{\left(0.0408^{{}^{\circ}}\right)}^{2}]$, respectively. It can be seen that the azimuth accuracy of $R^{u}(k)$ is far higher than the one of $R^{s}(k)$, but it will have numerous ambiguous estimates. The total tracking time is 10 s, with a sampling time interval 0.01 s.

In order to evaluate the performance of the tracking filter, root-mean-square error (RMSE) in position and azimuth are presented as:

(44) $$RMSE_{POS}\left(k\right)=\sqrt{\frac{1}{MC}\sum_{m=1}^{MC}{\left[{\hat{x}}_{m}^{u}\left(k|k\right)-x\left(k\right)\right]}^{2}+{\left[{\hat{y}}_{m}^{u}\left(k|k\right)-y\left(k\right)\right]}^{2}+{\left[{\hat{z}}_{m}^{u}\left(k|k\right)-z\left(k\right)\right]}^{2}}$$

(45) $$RMSE_{AZI}\left(k\right)=\sqrt{\frac{1}{MC}\sum_{m=1}^{MC}{\left[{\hat{\theta }}_{m}^{u}\left(k|k\right)-\theta \left(k\right)\right]}^{2}}$$

where, *MC* is the number of Monte Carlo simulations, $\left[\begin{array}{ccc} {\hat{x}}_{m}^{u}(k|k) & {\hat{y}}_{m}^{u}(k|k) & {\hat{z}}_{m}^{u}(k|k) \end{array}\right]$ and $\left[\begin{array}{ccc} x(k) & y(k) & z(k) \end{array}\right]$ are the state estimate and true value at time *k*, ${\hat{\theta }}_{m}^{u}(k|k)$ and θ(k) are the azimuth estimate and true value, respectively. Additionally, the subscript *m* means the index of Monte Carlo simulations. Correspondingly, the time-average RMSE (TARMSE) in position and azimuth can be further obtained by:

(46) $$TARMSE_{POS}=\frac{1}{L_{2}-L_{1}}\sum_{k=L_{1}+1}^{k=L_{2}}RMSE_{POS}\left(k\right)$$

(47) $$TARMSE_{AZI}=\frac{1}{L_{2}-L_{1}}\sum_{k=L_{1}+1}^{k=L_{2}}RMSE_{AZI}\left(k\right)$$

where *L*_1_ and *L*_2_ are the start and end time of evaluating the TARMSE.

**Figure 2:**
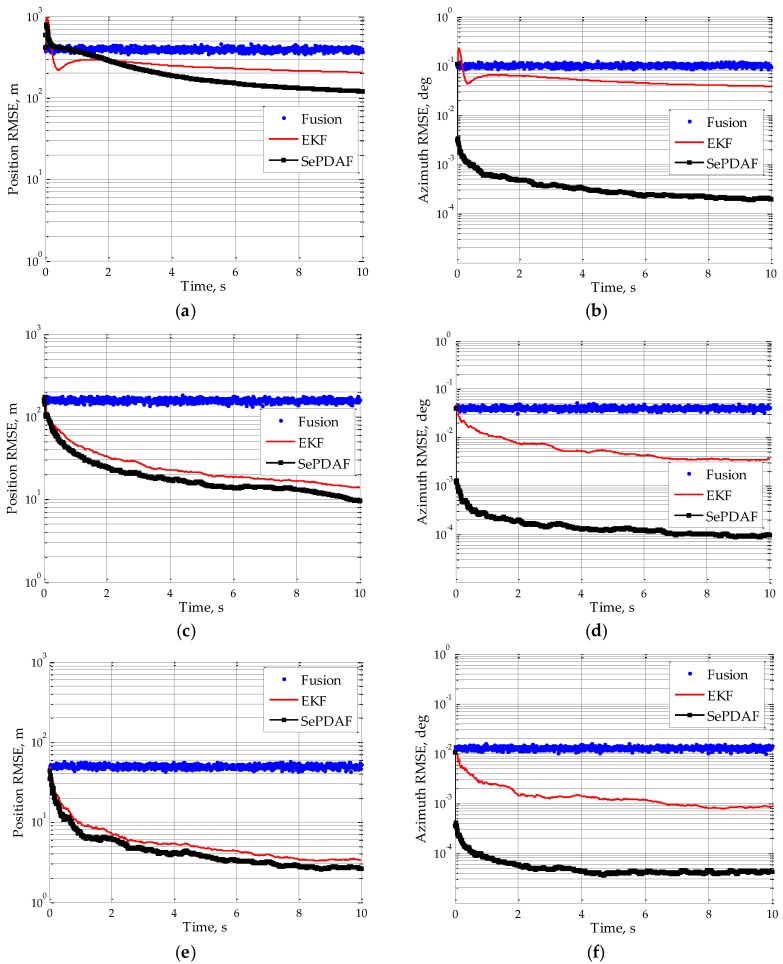
Position RMSE and azimuth RMSE after using the proposed method: (**a**,**b**) SNR = 12 dB; (**c**,**d**) SNR = 20 dB; and (**e**,**f**) SNR = 30 dB.

All the following simulations are carried out in MATLAB R2012b software (The MathWorks, Inc., Natick, MA, USA). Applying 100 Monte Carlo experiments, the position RMSE and azimuth RMSE under SNR = 12 dB, SNR = 20 dB and SNR = 30 dB by employing the proposed tracking method can be achieved, as shown in Figure 2. The original fusion measurement and corresponding EKF result using the traditional tracking mode are depicted as a comparison. It can be seen that the proposed algorithm will produce a stable trajectory and it will converge after 4 s. In addition, compared to the fusion result or the EKF result of the fusion measurement, the proposed SePDAF method will decrease the target position RMSE and significantly decrease the azimuth RMSE. That is to say, the proposed method could disambiguate the set of ambiguous angle estimates and acquire the high accuracy angle filtering result.

**Figure 3:**
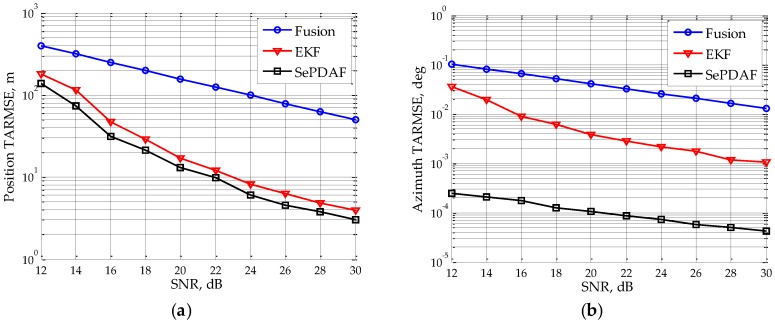
TARMSEs under different SNR after using the proposed method: (**a**) position RMSE; and (**b**) azimuth RMSE.

Assuming the SNR of single sub-radar varies from 12 dB to 30 dB with a step interval of 2 dB, then the target TARMSE of the position and azimuth under different SNR can be simulated, which is shown in Figure 3. It can be seen that with the SNR rising, the target TARMSEs of the position and azimuth will decrease gradually. Moreover, the azimuth TARMSE of the proposed method is reduced by a factor of about 1/25 when SNR is 30 dB, thus obtaining the high accuracy azimuth filtering result from the ambiguous estimates.

**Figure 4:**
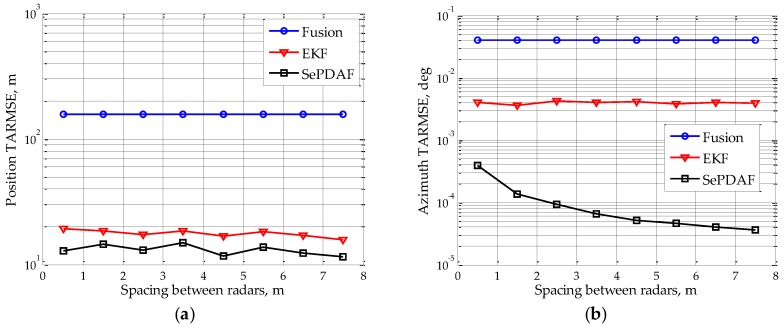
TARMSEs under different sub-radar spacing after using the proposed method: (**a**) position RMSE; and (**b**) azimuth RMSE.

Suppose the single sub-radar’s SNR is 20 dB, the sub-radar spacing changes from 0.5 m to 7.5 m with a step interval of 1 m. Figure 4 draws the target TARMSEs of position and azimuth under different sub-radar spacing. It indicates that the position TARMSE is almost identical with the sub-radar spacing rising, and it is the same for the fusion measurement and the EKF result. However, the azimuth TARMSE of the proposed method will drop greatly by a factor of 1/100 when the sub-radar spacing is increased to 7.5 m, thus achieving the high accuracy azimuth filtering estimate. The reason is that the increase of spacing can only improve the azimuth accuracy, while the range and elevation angle accuracy remain the same; thus, the position accuracy will not greatly improve.

**Figure 5:**
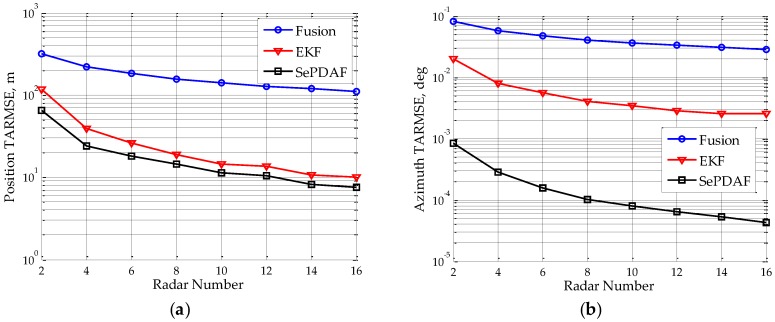
TARMSEs under different sub-radar number after using the proposed method: (**a**) position RMSE; and (**b**) azimuth RMSE.

In addition, assume the sub-radar number varies from 2–16 with a step interval of 2. Figure 5 depicts the target TARMSEs of position and azimuth under different sub-radar numbers. It denotes that the two TARMSEs will decline as the sub-radar number increases. Furthermore, the azimuth TARMSE will drop to 1/60 when the distributed array radar has 16 sub-radars. This indicates that the high accuracy true angle estimate has already been disambiguated from the ambiguous ones.

## 5. Conclusions

This paper proposes a second probability data association filter-based tracking method for distributed array radar. It firstly uses the fusion measurement of all radars to the EKF to achieve the first filtering. Then the SePDAF is applied as the second filtering which takes the first filtering result as prior knowledge and utilizes the array processed ambiguous angle measurements. Therefore, the target can be tracked steadily. Specifically, the azimuth filtering accuracy will be promoted significantly and the position filtering accuracy will also improve after employing this method. Additionally, this method has relatively low computational complexity. Finally, simulation results verify the effectiveness of the proposed method. Future works will concentrate on the verification of the proposed method in the real environment and even in the real battlefield by using an experimental radar system.

## Figures and Tables

**Table 1:** Computational complexity of EKF and SePDAF.

Operation	Complexity	EKF	SePDAF	SePDAF/EKF
$A_{N\times N}\times B_{N\times N}$	$O(N^{3})$	8	8	1.1
$A_{N\times N}^{-1}$	$O(N^{3})$	1	1	
|A|	$O(N^{3})$	0	1	
$A_{N\times N}\times B_{N\times 1}$	$O(N^{2})$	2	*m_k_* + 1	0.4*m_k_* + 0.9
$A_{N\times 1}\times B_{1\times N}$	$O(N^{2})$	0	*m_k_* + 1	
$A_{N\times N}+B_{N\times N}$	$O(N^{2})$	3	*m_k_* + 3	
$A_{N\times N}^{T}$	$O(N^{2})$	3	2	
$A_{1\times N}\times B_{N\times 1}$	O(N)	0	*m_k_*	2.5*m_k_* + 0.5
$A_{N\times 1}+B_{N\times 1}$	O(N)	2	2*m_k_*	
$\alpha \times A_{N\times 1}$	O(N)	0	*m_k_*	
$A_{N\times 1}^{T}$	O(N)	0	*m_k_* + 1	
$\exp(\beta_{n})$	$O(n^{2}\log n)$	0	*m_k_*	0.5*m_k_* + 1
$\tan^{-1}(\beta_{n})$	$O(n^{2}\log n)$	2	2	
$\beta_{n}\times \gamma_{n}$	$O(n^{2})$	31	2*m_k_* + 32	0.1*m_k_* + 1.1
$\sqrt{\beta_{n}}$	$O(n^{2})$	4	5	
$\beta_{n}+\gamma_{n}$	O(n)	6	*m_k_* + 5	0.2*m_k_* + 0.8

**Table 2:** Simulation parameters.

Parameter	Value	Parameter	Value
Frequency band	C-band	Carrier frequency (MHz)	6000
Sub-radar number	8	Bandwidth (MHz)	10
Sub-radar spacing (m)	2	Snapshot number	10
Baseline (m)	14	Process noise variance	1
Antenna elements number	20	Tracking time (s)	10
Antenna aperture (m)	0.5	Sampling time interval (s)	0.01
